# A call for better validation of opioid overdose risk algorithms

**DOI:** 10.1093/jamia/ocad110

**Published:** 2023-07-10

**Authors:** Duncan C McElfresh, Lucia Chen, Elizabeth Oliva, Vilija Joyce, Sherri Rose, Suzanne Tamang

**Affiliations:** Department of Health Policy, Stanford University, Stanford, California, USA; Program Evaluation Resource Center, Office of Mental Health and Suicide Prevention, US Department of Veterans Affairs, Menlo Park, California, USA; Department of Health Policy, Stanford University, Stanford, California, USA; Program Evaluation Resource Center, Office of Mental Health and Suicide Prevention, US Department of Veterans Affairs, Menlo Park, California, USA; Program Evaluation Resource Center, Office of Mental Health and Suicide Prevention, US Department of Veterans Affairs, Menlo Park, California, USA; Health Economics Resource Center, US Department of Veterans Affairs, Menlo Park, California, USA; Department of Health Policy, Stanford University, Stanford, California, USA; Program Evaluation Resource Center, Office of Mental Health and Suicide Prevention, US Department of Veterans Affairs, Menlo Park, California, USA; Department of Medicine, Stanford University, Stanford, California, USA

**Keywords:** clinical decision support, artificial intelligence, predictive modeling, algorithmic safety, opioid use disorder

## Abstract

Clinical decision support (CDS) systems powered by predictive models have the potential to improve the accuracy and efficiency of clinical decision-making. However, without sufficient validation, these systems have the potential to mislead clinicians and harm patients. This is especially true for CDS systems used by opioid prescribers and dispensers, where a flawed prediction can directly harm patients. To prevent these harms, regulators and researchers have proposed guidance for validating predictive models and CDS systems. However, this guidance is not universally followed and is not required by law. We call on CDS developers, deployers, and users to hold these systems to higher standards of clinical and technical validation. We provide a case study on two CDS systems deployed on a national scale in the United States for predicting a patient’s risk of adverse opioid-related events: the Stratification Tool for Opioid Risk Mitigation (STORM), used by the Veterans Health Administration, and NarxCare, a commercial system.

## INTRODUCTION

Clinical decision support (CDS) systems have played a role in medical decision-making for decades, and a wide variety of CDS systems are in use today.^1^^,^^2^ Many CDS systems are based on predictive models that estimate the risk of an adverse outcome, including complex machine learning methods such as artificial neural networks.^3^^,^^4^

Despite their growing use among US healthcare providers and health insurance companies, the impact of CDS systems on patient outcomes or costs is difficult to measure and there is conflicting evidence of their benefits to both patients and providers.^2^^,^^5^

We argue that technical validation and clinical validation are especially important for preventing harm. *Technical validation* addresses whether a CDS system meets its technical specifications such as prediction accuracy and software reliability; *clinical validation* addresses whether the system yields its intended impact on patients and providers. Researchers and regulators have proposed guidance for technical validation and clinical validation—including instructions for developing, implementing, and evaluating predictive models.^6^^,^^7^ The US Food and Drug Administration (FDA) has provided high-level guidance for the development and evaluation of Software as a Medical Device (SaMD), which includes CDS systems.^8^^,^^9^ While the FDA has not yet enforced this guidance, they have identified some CDS systems for future regulation. This includes one of the two prediction models we use as exemplars, which influences opioid prescription and dispensation decisions.^10^

Despite abundant guidance, many proposed and deployed CDS systems have not been technically and clinically validated by their developers or deployers and cannot be externally validated by others because there is insufficient information about their design and use.^11^ Furthermore, some systems are concealed as intellectual property or use “black-box” models that are not easily interpreted.^12^ This is concerning from a health equity perspective in that CDS systems have been found to discriminate against certain subgroups of patients.^13–20^

### CDS systems and the opioid epidemic

In 2021, there were 106 699 drug overdose deaths in the United States, most of which involved an opioid.^21^ In response to the opioid epidemic, many CDS systems have been proposed for estimating a patient’s risk of overdose, though few have been validated.^22^ We focus on two such systems: the Stratification Tool for Opioid Risk Mitigation (STORM), used by the Veterans Health Administration (VHA) and the Defense Health System, and NarxCare, a commercial system. Both systems are deployed on a national scale in the United States, and both use models that aim to predict risk of adverse opioid-related outcomes.

**Figure 1.**
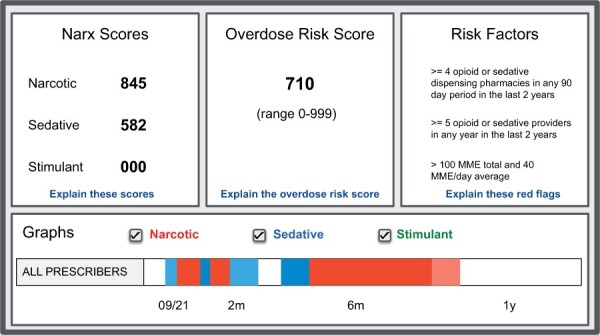
An illustration of the NarxCare interface based on NarxCare marketing materials.^23^ Top panels show various risk scores and risk factors. The Overdose Risk Score (center panel) estimates the patient’s risk of accidental death due to opioid overdose. Lower panels summarize the patient’s Prescription Drug Monitoring Program data.

*NarxCare*. In the early 2000s, US federal and state government agencies began surveilling controlled substance prescriptions in response to the accelerating opioid epidemic. Federal agencies encouraged states to adopt *Prescription Drug Monitoring Programs* (PDMPs), enabled by prescription databases of controlled substance prescription data. PDMP databases were made available to prescribers, dispensers, and law enforcement and allowed patient-level queries of prescription drug-seeking behaviors.^24^ One product of these efforts is the platform now known as NarxCare, which is maintained by Bamboo Health (https://bamboohealth.com, last accessed June 21, 2023).^24^^,^^25^ NarxCare’s interface (Figure 1) presents aggregated PDMP data, and risk scores based on these data, geared toward opioid prescribers and dispensers (https://bamboohealth.com/solutions/narxcare/, last accessed June 21, 2023). We focus on the NarxCare *Overdose Risk Score* (ORS), a number between 0 and 999 that indicates a patient’s risk of unintentional overdose death.^26^

**Figure 2.**
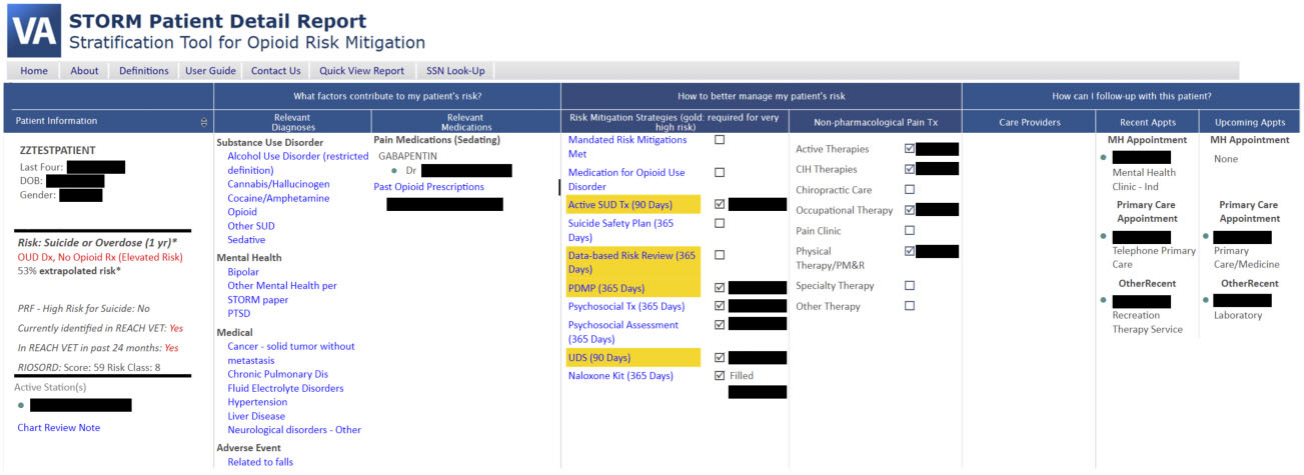
The STORM dashboard includes patient information including a risk score and risk factors (left columns), suggested risk mitigation strategies (middle), and treatment history and other provider information to facilitate care coordination (right). STORM: Stratification Tool for Opioid Risk Mitigation.

*STORM* is a model developed by the VHA for predicting risk of suicide- or overdose-related events. The VHA patient population has an especially high risk of opioid-related adverse outcomes,^27^^,^^28^ and STORM was developed as part of the VHA Opioid Safety Initiative (https://www.va.gov/PAINMANAGEMENT/Opioid_Safety, last accessed June 21, 2023). The STORM dashboard (Figure 2) displays a patient’s estimated risk score (a percentage of between 0% and 100%), risk factors identified by the model, information to coordinate with other care providers, and patient-tailored clinical guideline recommendations for risk mitigation strategies to be considered in the patient’s treatment plan.

Although STORM and NarxCare have a similar function, their approach to validation is very different. Evaluations of NarxCare-ORS focus only on technical validation—specifically, they evaluate the predictive performance for a narrow patient cohort, and do not address its impact on patients or providers. In contrast, STORM has been validated from both a technical and clinical perspective.

We are not the first to raise concerns about NarxCare or other CDS systems that are poorly validated. Oliva^25^ warns that using NarxCare and PDMP data to guide prescribing and dispensing decisions can harm patients with complex pain conditions, uninsured or underinsured patients, and those with co-morbidities or disabilities. The California Society of Addiction Medicine raised concerns of NarxCare’s transparency and insufficient validation.^29^ Kilby^30^ argues that, in general, predictive models related to opioid use do not align with the goal of improving patient care. In a WIRED article, Szalavitz^31^ reports on individual patient experiences and instances of harm associated with NarxCare, suggesting that it may pose safety and effectiveness issues.

We cannot determine how, or if, NarxCare can be fixed. Rather we intend this case study as a call to action for CDS developers, deployers, and users, to hold CDS systems to higher standards of validation.

## VALIDATING CDS SYSTEMS AND PREDICTIVE MODELS

We separate the validation process into two steps: (1) defining a system’s purpose and intended impact and (2) evaluating it from a clinical and technical perspective.

### Purpose and intended impact

To validate a CDS system we first need to answer the questions: *which healthcare decisions is this tool meant to influence, and how?* and *what is the intended impact on patients and providers?* FDA SaMD guidance suggests that CDS developers answer these questions in a *definition statement*,^8^ which underpins the entire SaMD review framework. This statement tells regulators how to evaluate a tool’s performance, its intended use, and the severity of potential impacts on patients.

#### NarxCare-ORS: purpose and impact

Public documents provide conflicting definitions of NarxCare’s purpose. Marketing materials state that ORS scores are only intended for “raising awareness,” and not as a basis for clinical decisions—suggesting that NarxCare and ORS are not intended for CDS.^23^ Yet, NarxCare user guides provide explicit recommendations for how ORS scores should be interpreted and used for clinical decision-making.^32^ Furthermore, no documentation defines the clinical or operational outcomes that NarxCare is meant to impact—such as overdose rate, naloxone dispensation and prescription, or referrals to pain or addiction specialists.

This ambiguity is problematic: the manufacturer states that NarxCare is not intended for CDS; yet, NarxCare is currently used by (and marketed toward) many care providers across the United States (https://bamboohealth.com, last accessed June 21, 2023). Furthermore, PDMP data directly influence clinicians’ treatment decisions,^33^^,^^34^ so we should expect that PDMP-derived ORS scores will have a similar influence on clinicians.

#### STORM: purpose and impact

The STORM developers state that STORM risk scores are intended to help providers prioritize patients for case review and intervention.^35^ In a clinical study protocol, Chinman et al^36^ state that providers should conduct case reviews for patients categorized in the top tier of risk scores and describe several key outcomes for validation—including clinical outcomes (overdose- and suicide-related events, accidents) and implementation outcomes (number of case reviews and opioid mitigation strategies).

### Technical and clinical evaluations

*Technical evaluation* addresses questions of the underlying technology. One aspect of technical evaluation is software reliability, IT integration, and compliance with stated requirements.^9^^,^^37^^,^^38^ Another aspect assesses the generalizability of predictive models and identifies potential disparities in performance for patient subgroups and historically marginalized groups.^39–41^ These evaluations are typically conducted prior to deployment, often using simulations.^42^

*Clinical evaluation* assesses a system’s impact on patients and providers. This includes measuring health and operational outcomes and determining whether the system is used correctly according to the manufacturer’s instructions.^9^ Unlike technical evaluation, clinical evaluation usually requires a trial or pilot study.

#### NarxCare-ORS: evaluation

There are three public studies of NarxCare-ORS that use a small cohort of patients from Ohio, Indiana, and Michigan, which find that NarxCare-ORS is somewhat predictive of opioid-related outcomes and is comparable with simpler “red flag” methods of risk assessment.^43–45^ Other studies find that ORS scores are predictive of non-opioid-related outcomes not related to NarxCare’s intended use.^46–48^

These evaluations are insufficient for several key reasons. First, these studies consider a narrow cohort: they include a subset of patients in Ohio, Michigan, and Indiana. While ORS scores are somewhat predictive of opioid-related outcomes for these patients, this finding does not necessarily generalize to other states. This is troubling because NarxCare is reportedly used by 44 of the 54 PDMP programs in the United States and in all 50 US states (https://bamboohealth.com/audience/state-governments, last accessed June 21, 2023). In other words, NarxCare-ORS has not been technically validated for *the vast majority* of patients who are scored by this tool. Second, there has been no subgroup analysis of ORS scores. The predictive utility of ORS may vary depending on demographics, insurance status, and access to healthcare.^39^

Third, and most importantly, NarxCare-ORS has *never been clinically evaluated*. For NarxCare-ORS, a clinical evaluation might measure impacts on health indicators such as opioid overdose rate, mortality, and naloxone use—and provider-related indicators such as adherence to manufacturer guidelines, number and type of prescriptions, and patient referrals.

#### STORM: evaluation

There have been one technical evaluation and several clinical and implementation evaluations of STORM. An initial technical evaluation was conducted by STORM developers using a cohort of 1 135 601 patients—including almost all VHA patients who had an opioid prescription in 2010.^35^ Unlike NarxCare-ORS evaluations, this study includes a cohort of patients that is nationally representative of the patients that STORM is intended for: all VHA patients who received an opioid prescription from VA.

STORM clinical evaluations incorporated strong randomized evaluation design to measure the impact on opioid-related health outcomes,^49^^,^^50^ strategies for integrating STORM into clinical workflows,^51^ and oversight of STORM use.^52^^,^^53^ These studies examined the clinical impact of use of the STORM CDS system to target a prevention program. This included effect on clinical outcomes and assessment of change in clinical process and practice.

## DISCUSSION

In the context of the opioid epidemic, an improperly validated CDS system can have severe consequences. For example, if the NarxCare model leads a pharmacist to believe that a patient’s risk of overdose is higher than their actual risk, the pharmacist may decline to fill the patient’s prescription, disrupting their treatment plan. This can leave the patient frustrated and with potentially disabling pain due to poor model design choices such as those described in the gray literature.^31^ A model can also suggest that a patient’s risk is lower than their actual risk and miss an opportunity to prevent opioid misuse or a lethal overdose. Model errors like this are impossible to avoid in practice. Furthermore, data in healthcare settings are collected for the purpose of supporting administrative billing and regulatory reporting. They are a collection of observations about a patient when they are sick, not healthy, and often plagued by missing values, selection bias, confounding, irregular sampling and temporal drift—making it difficult to draw generalizable conclusions.^54^ There are also widely known challenges with the prediction of rare events.

Improper *use* of predictive models can be just as harmful as deploying faulty models. PDMP data influence prescription decisions in a variety of ways: some providers use these data as a justification to refuse prescription or treatment, or even discharge patients from a practice.^33^^,^^34^ We should expect that some clinicians will use NarxCare-ORS—a PDMP-derived risk score—to refuse treatment for certain patients. And manufacturer guidance here is contradictory: marketing materials state that ORS should not influence prescription decisions,^23^ while user guides recommend specific prescriber actions corresponding to ORS score ranges.^32^

In contrast, STORM’s intended use and intended outcomes were defined and validated during development and evaluation.^35^^,^^36^^,^^49–53^ While STORM and NarxCare include risk scores, STORM recommends risk mitigation strategies based on clinical guidelines, and information to facilitate care coordination.

While it is useful to contrast NarxCare-ORS with STORM, both systems have limitations. The STORM model has only been updated once (to a 2014–2015 cohort) since its original development on a 2010–2011 cohort^35^ and should be re-evaluated and updated using more recent data. Furthermore, the developers have identified subgroup disparities in STORM indicating worse predictive performance for veterans who are older, non-white, and female^55^^,^^56^; ongoing work aims to mitigate these disparities. There is no public subgroup analysis of NarxCare, though performance disparities can potentially harm marginalized groups.^25^

Regulators and researchers provide guidance for CDS system validation, but this guidance is not universally followed by CDS developers and users and is not enforced by regulators. For this reason, healthcare providers in the United States *cannot assume that CDS systems have been validated*, and healthcare systems should establish oversight procedures to independently ensure that these tools satisfy basic requirements of safety and quality. Some academic healthcare systems have started this effort, including Duke Medical Center (https://aihealth.duke.edu/algorithm-based-clinical-decision-support-abcds/, last accessed June 21, 2023), University of Wisconsin Health,^57^ and Stanford Medicine.^58^ These programs can serve as an example for others, though it is important to acknowledge that each provider is different: there is no “one-size-fits-all” approach to algorithmic governance.

The current environment also poses challenges to CDS developers, as both governance standards and FDA SaMD guidance continue to evolve. There are no formal mechanisms to enforce requirements on SaMD applications, and current guidance does not explicitly define requirements (such as criteria for transparency or validation). For this reason, CDS developers face enormous uncertainty about *what* these regulations will require, and *when* and *how* they will be enforced.

As standards for algorithmic governance in healthcare begin to take shape, we encourage the creators, deployers, and users of CDS systems to focus not only on their technical capabilities but also on their purpose, intended use, and most importantly, their impact on patients.

## Data Availability

No data were generated or analysed in support of this research.
